# Urban Public Sports Information-Sharing Technology Based on Internet of Things

**DOI:** 10.1155/2021/5438584

**Published:** 2021-12-08

**Authors:** Youliang Li, Fenglei Li, Yujun Xiong

**Affiliations:** School of Physical Education, East China University of Technology, Nanchang 330013, China

## Abstract

With the continuous development of social economy, sports has become one of the important ways of physical exercise, and the demand for corresponding sports facilities is also increasing. The Internet of Things technology is introduced in this paper. Through combining the current status of urban public sports, an urban public sports sharing system is built by trial, to promote the sharing of urban public sports information through the continuous development of new technologies such as the Internet and improve the publicity and popularization of public sports information. Simulation experiments prove that the Internet of Things technology is effective and can effectively support the sharing of urban public sports information.

## 1. Introduction

With the continuous development of social economy, modern society has gradually transformed into a “data society,” and all walks of life are subject to important influences and innovations related to informatization [1, 2]. The continuous progress of society has promoted the healthy development of human beings, but how to conduct sports or physical exercise is an issue that people are extremely concerned about, especially about the existence of surrounding stadiums and public welfare sports facilities, vacant venues for the badminton courts, etc. [3, 4].

From digital cities to smart cities, the construction of informatization has had a profound impact on various industries, and data have also grown in an explosive manner. However, disorderly and messy data often bring about important loads of hardware storage and network transmission and have become a burden on users [5, 6]. However, what needs to be valued is how to reasonably process, orderly process, and classify and extract these data, realize the reuse of data, tap its potential value, transmit potential information, realize the maximum sharing of data and information, and save the cost of data and information and various construction costs [7, 8]. The continuous construction and development of smart cities have gradually introduced a series of new technologies and methods such as the Internet of Things, the Internet, CloudNative, and Fog Computing, aiming to solve urban sickness such as urban congestion, environmental pollution, population agglomeration, and other cities in the process of urbanization [9, 10].

For sports, it is necessary to further consider its limitations and needs in the actual implementation process, how to select the location of sports facilities, how to release it to the public, when will the corresponding venue information be opened, how many visitors access the venues currently in real time, and so on [11, 12]. According to the different seasons, some information is extremely valued such as opening college swimming pools and badminton halls to the public, related matters, and public opening hours, to prevent the public from entering disorderly. In response to these needs and limitations, the new technologies such as the Internet of Things is introduced in this paper, and an urban public sports information-sharing architecture system is built by trial. Through the Internet and other technologies, information is continuously processed, extracted, and shared, and finally, information sharing is realized, aiming to improve the quality of public sports, realizing “one-time processing and analysis, and multiple times' sharing applications.”

## 2. The Status Quo of the Co-Construction and Sharing of Sports Information Resources in China

The advent of the information age has given birth to the increase and enrichment of various information resources. As far as the sports industry is concerned, its information resources are an important branch of related information resources, which have the extensiveness and imbalance of traditional information resources. Similarly, sports resource information still contains a lot of content, but the quality is difficult to be guaranteed. Because sports has penetrated every walk of life, such as sports channels, sports pages of web news, sports sections in micromedia, sports bloggers, and various sports magazines, there are various carriers of sports information resources, even new media such as Douyin and Kuaishou, have a large amount of sports information resources. This aspect is due to the continuous development of information technology and the continuous enrichment of carriers. On the contrary, sports have become the focus of daily life and gradually attract more and more people's attention. How to provide important sports information to the public has become a problem and direction worthy of attention in the industry and academia [13, 14]. Therefore, it is necessary for all sports-related industries to work together to improve business capabilities and realize the sharing and co-construction of sports resources.

### 2.1. Outdated Concepts and Weak Consciousness

As a public welfare direction, usually sports does not receive too much guarantee in terms of policies, especially funding guarantees; it is difficult to be guaranteed completely. According to the research statistics of relevant experts, in the sports industry, construction of informatization for the related sports scientific research projects is hardly valued. On the one hand, the construction of sports facilities requires a high cost; on the other hand, it is difficult to keep up with management concepts and awareness. The relevant expert often meets with the trouble in calling for the strengthening of the co-construction and sharing of related information-based sports resources. On the one hand, information-based resources are less; even if they are shared, they cannot meet the requirements; on the other hand, institutions engaged in corresponding sports scientific research have few projects of related research and conducted few informatization research studies [15, 16]. In addition, managers in the actual sports industry still cannot have this kind of thinking or courage to realize the direct sharing of relevant information. On the one hand, the requirement and support from the relevant industry authorities are lacked. On the other hand, most industry managers believe that they have no obligation to provide information resources to other units. This has great limitations on information management and urban management, and it is not conducive to the sharing of sports resource information for urban residents.

### 2.2. Barriers Exists and Each Does Things in His Own Way

As far as the administrative authorities are concerned, the resource information is controlled by the corresponding competent authority; the physical education colleges for college students have relevant information resources, so the corresponding sports information center, scientific research institute, or university sports library are all carrying out their respective construction of informatization, in which there are not only technical gaps in the underlying system development but also differences in operating networks, all of which cause more trouble for information sharing to form an information barrier [17, 18].

### 2.3. Lack of Talents, Low Development, and Integration Capabilities

For a long time, because the construction and development of sports informatization have not been valued correspondingly, there are fewer composite talents with related sports knowledge in the field of sports informatization, including computer network technology, especially the high-quality sports services are less such as technical consultation sports characteristic databases and network academics [19, 20].

## 3. Conditions for Constructing a Platform for the Co-Construction and Sharing of Sports Information Resources in China

### 3.1. The Continuous Growth of Sports Digital Resources Provides a Resource Base

With the continuous development of the social economy, the sports information resource database has also been continuously enriched, which lays a solid foundation for the construction of a corresponding sports information resource-sharing platform. With the use of networked and electronic related technologies, the more demand emerges to use a unified portal for unrestricted access to corresponding information resources. Therefore, it is extremely important to establish corresponding sports shared information service platform. The continuous development of computer technology and network transmission technology can realize the integration of resources and one-stop fast service.

### 3.2. Coordination of Supply Entities

For most entities, the governance of collaborative information becomes possible because public sports services can involve multiple objects, such as the government, users, and markets, and require multiple parties to conduct collaborative management, which is mainly reflected in multiple aspects, such as under the background of informatization; multiple entities realize the supply and service of sports public services and realize the improvement of the overall quality of urban sports public services. Ensure that the form of government tends to be flat vertically; horizontally, we achieve coordination, unification, and cooperative management and governance between governments, focusing on solving the problem of fragmentation.

In addition, big data technology can ensure that online sports services are possible and clarify the further enhancement of the entity service. The traditional sports supply is managed by different administrative departments according to different responsibilities, giving full play to the advantages of technology and knowledge, so that all aspects are all in the state of co-construction and sharing. However, what needs to be valued is that various factors such as the positions of various units, the cost of information sharing, and the different technical standards have caused various departments to form different service models. Therefore, the corresponding departments should be linked to realize the virtual cooperation in demand for sports public services, to ensure that the individual's management and service capabilities are improved, to achieve a more fragmented and detailed public sports services, to achieve the refinement of the tasks of the public service entity, to remove the traditional steps designated by the higher level, to optimize the organizational relationship, and to achieve flattening.

In the era of big data, there is a clear gap between the public and the actual supply of sports services. On the one hand, the public cannot accurately express the demand for sports facilities, and on the other hand, there is a structural dislocation in the demand for sports public services.

Before the era of big data, there is often a clear gap between people's expectations for public sports services and the supply of public sports services. The reasons are that the public's demand for public sports services is not fully and clearly expressed, the government's decision-making model is not sufficient, and there is a structural dislocation between the demand for sports public services and the supply. In the era of big data, public sports services are becoming more and more inventory oriented. The total public demand for sports and configuration needs of the people are clearly expressed, and they are realized through the intelligent perception of sports public service needs and service data decision-making.

First of all, the intelligent perception of sports public service needs. To fully and clearly express the people's sports service needs, the government can increase interaction with the society through open methods and can also use big data technology to intelligently acquire and perceive the people's public sports needs. The mainstream web3.0 technology of big data solves the inaccessibility of people's information technology and greatly reduces the cost of information and data transmission. The public can also use various terminals such as websites, Weibo, WeChat, and application software to express their needs for sports services; the government uses intelligent-sensing technology to gather these microdata reflecting people's needs and aggregate them into the big data needed for statistics. Relying on big data analysis and mining technology, data are processed and people's specific needs are analyzed for sports public services. Second is data decision-making for the provision of sports public services. The total amount of services and the allocation of resources are the main content involved in the supply of sports public services; in the final analysis, it is a matter of decision-making. At present, the supply of sports public services is not balanced. The key factor is that the government's decision-making model is not perfect; the factor that the government's decision-making model is not perfect is that the decision-making body often makes decisions based on their own interests and preferences and does not fully consider the people's sports needs; with comparison of decision-making methods, subjectively, it is difficult for the government to make scientific decisions in the absence of samples and data information. The use of big data largely solves the problems of data complexity, uneven levels, and in-depth data analysis. Data-driven decision-making has gradually become an important model for the precise supply of sports public services. Third, the supply of public sports services tends to be inventory based. The supply of sports public services covers the overlap between supply and demand. The supply is related to the financial situation of the local government, the level of social and economic development, and the realization of government functions. The cloud platform can be used to systematically integrate. The big data of the database are sorted out, and the supply of sports public services is accurately mined with the help of MapReduce technology. Demand refers to the type and quantity of people's needs for sports public services, which requires big data technology to collect social information more intelligently and comprehensively, such as through websites, Weibo, WeChat, and other terminals. With the help of big data technology and analysis technology, realize mining and evaluation.

With the help of big data resources and technology, we can effectively predict the total amount of sports public services and optimize their configuration to alleviate the problem of unbalanced supply on the demand side. At the same time, big data have also become an efficient and intelligent tool for the main body (government) on the supply side of sports public services, making the supply of sports public services more intelligent, precise, and simple, thereby realizing the government's modern governance goals.

First, the supply of sports public services is smarter. First of all, the auxiliary equipment for sports public services has realized intelligence. Big data promote APP-based terminal applications. By integrating government websites and government service AGM and other auxiliary equipment, the intelligent model can be used to reach all distances of sports public services. Secondly, the willingness to supply sports public services has become intelligent. Before the era of big data, government websites were mainly based on supply-oriented service models that only responded to requests. This model was relatively passive, and government supply usually did not match the people's sports needs. In the era of big data, the government can use online analysis tools such as Google Analytics, Urchin, and SiteCastalyst to collect and analyze data information such as the types and characteristics of search keywords and use Hadoop and other tools to analyze people's search behaviors, effectively based on people's points of interest. Push the corresponding sports service. Second, the supply of sports public services is more precise. Individual needs in the era of big data have become the focus of attention of enterprises, society, and government. Big data advocate the concepts of information openness, sharing, fairness, and information decision-making. Its mining analysis technology not only analyzes the sports public service database but also effectively analyzes the data of the Internet and mobile terminals and uses natural language decryption software to analyze unstructured data. The data can be used to identify the potential actions of users, so as to provide targeted sports services for them, and “let the data speak” so that both the supplier and the demander can see the visualized results. Third, the supply of sports public services is more concise. Before the big data era, the supply of sports public services was based on the division of labor based on professionalism, and the operation process was constructed in the form of bureaucracy. In this way, a very complete sports public service chain was divided into countless broken links to make its service process more messy. The fragmented work process caused not only delays in service work but also an embarrassing situation in which operating costs exceeded benefits. Integrating resources is the important significance of the existence of big data, and the supply of sports public services under big data should also be transformed from the traditional multichannel model to a simplified model, integrating the application layer, data layer, and platform layer of different government functions in the background. In order to better provide resources and system support for the front end, we build an application system at the application layer to achieve connection and communication, establish a government department database at the data layer and open data information, establish a platform for government business support at the platform layer, and integrate sports at the front end. The public service client is integrated to provide a way of integration for the background. Unify the website port with the mobile port to provide simplified public sports services.

### 3.3. Principles of Sports Information Resource Sharing

Assume that the subtask sample dataset of *n* platform data traffic shunt is

(1) $$\begin{array}{c} T\left(n\right)=\left\{t_{1},t_{2},\ldots ,t_{n}\right\}. \end{array}$$

Among them, *n* ∈ *N* and *t*_*i*_ represents the *i*th shunt subtask (*i*=1,2,…, *n*) in the set. The corresponding attribute vector of the shunt task *t*_*i*_ is expressed by

(2) $$\begin{array}{c} t_{i}=\left(t_{\text{id}}^{i},t_{\text{mi}}^{i},t_{\text{fee}}^{i},t_{\text{deadline}}^{i},t_{\text{memory}}^{i},t_{\text{bw}}^{i},t_{\text{submit}}^{i}\right). \end{array}$$

Among them, *t*_id_^*i*^ represents the unique tag number of the task, *t*_mi_^*i*^ represents the size of the platform data traffic task, which reflects the number of tens of millions of instructions (MI) of the task, *t*_fee_^*i*^ represents the cost of the user's desired task, *t*_deadline_^*i*^ represents the deadline of the user's desired shunt task, *t*_memory_^*i*^ represents the memory size requirement of the shunt task, *t*_bw_^*i*^ represents the bandwidth requirement of the shunt task, and *t*_submit_^*i*^ represents the time when the network user submits the shunt task.

Assume that the sample dataset of *m* virtual machine platform data traffic resources is

(3) $$\begin{array}{c} VM\left(m\right)=\left\{vm_{1},vm_{2},\ldots ,vm_{m}\right\}\left(m\in N\right). \end{array}$$

Among them, *vm*_*j*_ represents the *j*th virtual machine (*j*=1,2,…, *m*), to calculate the corresponding attribute vector of the virtual machine resource:

(4) $$\begin{array}{c} vm_{j}=\left(vm_{\text{id}}^{j},vm_{\text{capacity}}^{j},vm_{\text{bw}}^{j},vm_{\text{memory}}^{j}\right), \end{array}$$

where *vm*_id_^*j*^ represents the unique tag number of the data traffic data center of the platform where the virtual machine is located, *vm*_capacity_^*j*^ represents the processing capacity of the virtual machine resources, *vm*_bw_^*j*^ represents the bandwidth provided by the virtual machine, and *vm*_memory_^*j*^ represents the memory size of the virtual machine, and ETC_*ij*_ is utilized to reflect the expected execution time of platform data traffic shunt subtasks *t*_*i*_ on virtual resources *vm*_*i*_:

(5) $$\begin{array}{c} {\text{ETC}}_{ij}=\frac{t_{mi}^{i}}{vm_{\text{capacity}}^{j}}. \end{array}$$

Let *be*_*j*_ represent the initial time for execution on different virtual machines and ETC_*ij*_ represent the expected completion time of the shunt task executed on the virtual machine *vm*_*ij*_:

(6) $$\begin{array}{c} {\text{ECT}}_{ij}=be_{j}+{\text{ETC}}_{ij}. \end{array}$$

The completion time of all platform data traffic shunt tasks is denoted as Makespan; then,

(7) $$\begin{array}{c} \text{Makespan}=\max\left\{{\text{ECT}}_{ij}\right\}. \end{array}$$

Formula (8) is used to express the objective function and constraint conditions of the platform data traffic offload task:

(8) $$\begin{array}{c} \left\{\begin{array}{c} \min\left\{\text{Makespan}\right\},\\ \left\{\begin{array}{c} t_{\text{memory}}^{i}\leq vm_{\text{memory}}^{j},\;i=1,2,\ldots ,n,\\ t_{\text{bw}}^{i}\leq vm_{\text{bw}}^{j},\;j=1,2,\ldots ,m. \end{array}\right. \end{array}\right. \end{array}$$

In the form of linear programming, the objective function given by equation (8) is solved. Assuming that equation (8) is established, *ω*′ is the optimal solution for the current platform data traffic shunt task, and equation (9) is used to establish a cloud platform data traffic management model:

(9) $$\begin{array}{c} \left\{\begin{array}{c} \omega^{\prime }=\omega +\frac{\left(1-p\right)\left(1-\text{rand}\left(\right)\right)}{e^{y}},\\ {1/e}^{\Delta y/\left(1+T_{i}\right)}>\text{rand}\left(\;\right), \end{array}\right. \end{array}$$

where rand () is a random number in the interval [0, 1], *p* is the mutation probability, and *T*_*t*_ represents the completion time of different subtasks in the objective function constraint condition determined by Makespan.

Assume that the set of platform data traffic shunt subtasks composed of sample data of *k* platform data traffic shunt subtasks is

(10) $$\begin{array}{c} c={\left[c\left(0\right),c\left(1\right),\ldots ,c\left(e-1\right)\right]}^{k}. \end{array}$$

Among them, to satisfy the conditions of *a* ~ *g*(*c*, *s*), *g*(*s|c*) represents the distribution probability of subtasks in the shunt task, and we calculate the shunt task load of total platform data traffic:

(11) $$\begin{array}{c} \hat{s}=\varepsilon \text{ arc}\underset{q\in \varepsilon }{\min}\sum_{s=1}^{+\infty }H\left(\hat{s},s\right)g\left(s|c\right), \end{array}$$

where $H\left(\hat{s},s\right)$ represents the cost function in the shunt task and *ε* represents the set of all subtasks in the shunt task:

(12) $$\begin{array}{c} \varepsilon =\left\{\hat{s}|c_{1}+2c_{r}\leq \hat{s}\leq S\right\}, \end{array}$$

where *S* represents the maximum value of the task in the process of shunting tasks, and the cost function is calculated using

(13) $$\begin{array}{c} H\left(\hat{s},s\right)=\left(\hat{s},s\right)={\left(\hat{s}-s\right)}^{2}, \end{array}$$

where $\hat{s}$ represents the mean value of the time required for the shunting process. Assuming that the diversion time meets the condition of $\sum_{s=1}^{s}\hat{s}\left(s|c\right)=1$, the estimated function can be obtained:

(14) $$\begin{array}{c} H\left(\hat{s},s\right)=\left|\hat{s}-s\right|. \end{array}$$

Substitute formula (13) into (11), the following results can be obtained:

(15) $$\begin{array}{c} \hat{s}=\text{arc}\underset{s}{\min}\hat{s}\left(\sum_{s=1}s\left(s|s\right)-\sum_{s=s}^{s}s\left(s|c\right)\right). \end{array}$$

Realize the calculation of the amount of subtasks in the process of platform data traffic shunting:

(16) $$\begin{array}{c} s=\text{arc}\underset{s}{\max}\left(\bar{s}|c\right). \end{array}$$

In summary, the amount of subtasks in the platform data traffic offloading task can be calculated, and the dynamic priority task of the platform data traffic shunt task can be obtained, which provides an accurate data foundation for the establishment of the data traffic optimization management model of the big data cloud platform.

To ensure that sports information play the role of co-construction and sharing, therefore, a co-construction and sharing platform is built by trial to realize the sharing of platform information in this paper.

According to the experience of various successful examples, the co-construction and sharing system platform is based on a library with rich digital resources and power (information centers) as the “leader” in a region (or industry), which leads everyone in the construction.

**Figure 1:**
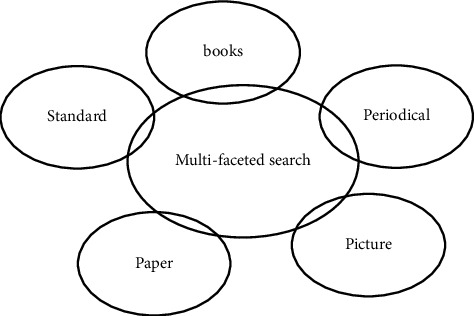
Searching and sorting out of existing digital resources.

The first is to integrate existing digital resources, including local mirroring resources and remotely accessible resources, as shown in Figure 1, such as Chinese and foreign language collection catalogs, e-books, e-journals, and video materials.

The second is the division of labor and cooperation. The corresponding characteristic database is established based on different data. If the corresponding information resource database is constructed according to the sports items, the corresponding sports resource database will be formed, and continuous incremental updates will be carried out.

## 4. Simulation Experiment

### 4.1. Overall Objectives

Based on the development needs of the city, starting from the sharing of urban public sports information technology, technologies such as the Internet and the Internet of Things are introduced as an emphasis, to realize the relevant sharing of sports information by building a corresponding technical platform.

### 4.2. Overall Thinking

**Figure 2:**
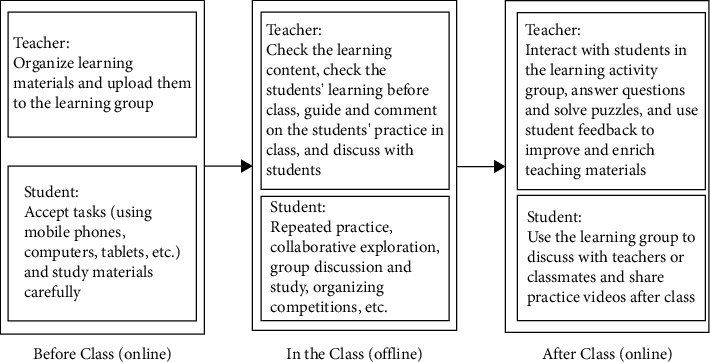
Procedure diagram of the online and offline mixed teaching mode of higher vocational physical education.

According to the urban public sports information sharing, the corresponding framework is realized, as shown in Figure 2.

In the higher vocational physical education class, the online and offline hybrid teaching mode is used, and the general process of using urban public sports information is carried out, as shown in Figure 2.

1. The first stage: online learning before class.
2. According to the educational programs, the teacher before class organizes the learning materials online and uploads them to the learning and exchange group.
3. The second stage: offline learning in class.
4. In the classroom, teachers can give targeted explanations based on students' preclass learning situation to improve classroom efficiency. Meanwhile, teachers should help the students carry out the explorative study, solve preclass questions, and consolidate knowledge and skills.
5. The third stage: online consolidation after class.
6. After class, teachers continue to interact with students in the online learning platform to help students solve problems encountered in the learning process. Meanwhile, teachers improve and enrich learning materials and improve teaching methods based on students' feedback.

### 4.3. Establishment of Database

**Figure 3:**
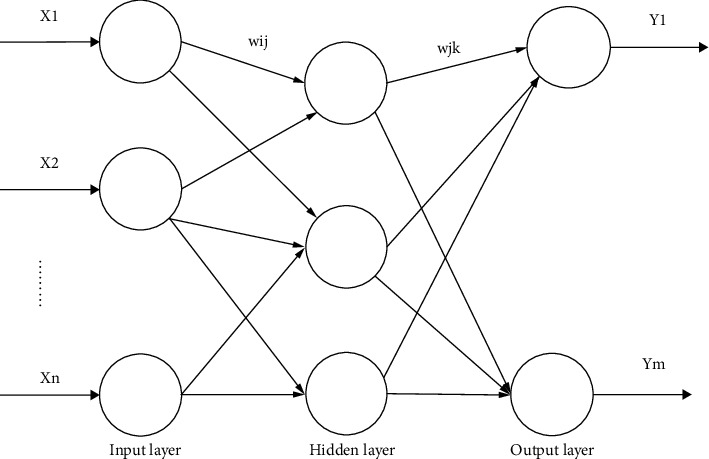
Topological structure diagram.

Using urban public sports information, the original data of 23,001 students from freshman to junior year are removed and cleaned and missing value data were purged, and data of 21,089 students were retained. A model of “the association between the physique test grade and each individual index” is established, as shown in Figure 3, to study the influence of each individual index of the physique test on the overall physical fitness assessment.

**Figure 4:**
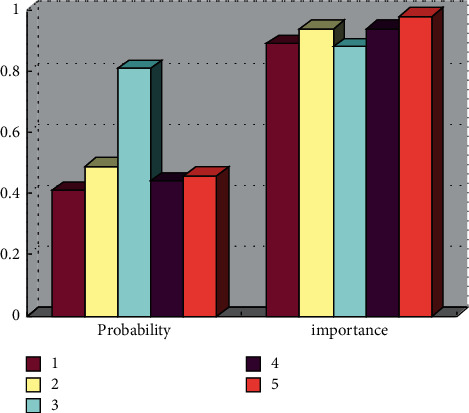
Some part of the results of association rule mining.

A large number of association rules are excavated, and the typical correlation rule is screened out, as shown in Figure 4, which will be beneficial to the decision support of this research goal.

41% of the senior girls are rated as with excellent physiques and with excellent speed and flexibility. Rule 2 shows that 48.5% of girls are rated as excellent with excellent speed and vital capacity and body mass index. Rule 3 shows that 82.4% of the senior girls who passed the overall evaluation score failed in speed, but had excellent flexibility, with an importance of 0.877025, indicating that even though the body has good flexibility, while the speed is poor, the overall evaluation results are only qualified. Rule 4 shows that the physical flexibility and BMI of the failed sophomore girls are both unqualified. The probability and importance are 0.44 and 0.94, respectively. It can be seen that the body shape and flexibility have a huge impact on the physical fitness test level. Rule 5 shows that even if the endurance score passes, the sophomore girl with poor vital capacity is unqualified and the probability and importance are 0.46 and 0.98, respectively. According to the comprehensive analysis of the results of urban public sports information sharing, girls with excellent physique evaluation have excellent speed, flexibility, and vital capacity. However, girls with poor physique evaluation are mainly caused by poor performance in speed, endurance, and vital capacity. Simulation experiments prove that technologies such as the Internet of Things and the Internet are effective and can support the sharing of urban public sports information.

## 5. Conclusions

With the advent of the era of big data, how to share urban public sports information has become an increasingly important issue. The status quo of the co-construction and sharing of sports information resources are sorted out by introducing related technologies such as the Internet of Things, the Internet, and artificial intelligence, and the needs and difficulties of co-construction and sharing of sports information resources are analyzed and constructed by trial in this paper. The aim is to promote the openness and popularization of public sports information by realizing the corresponding sharing scheme. Simulation experiments prove that the Internet of Things technology is effective and can effectively support the sharing of urban public sports information.

## Data Availability

Data sharing is not applicable to this article as no datasets were generated or analyzed during the current study.
